# Web GIS in practice VIII: HTML5 and the canvas element for interactive online mapping

**DOI:** 10.1186/1476-072X-9-14

**Published:** 2010-03-03

**Authors:** Maged N Kamel Boulos, Jeffrey Warren, Jianya Gong, Peng Yue

**Affiliations:** 1 Faculty of Health, University of Plymouth, Drake Circus, Plymouth, Devon PL4 8AA, UK; 2 MIT Media Laboratory, Design Ecology Group, Massachusetts Institute of Technology, 20 Ames Street, Cambridge, MA 02139, USA; 3 State Key Laboratory of Information Engineering in Surveying, Mapping and Remote Sensing (LIESMARS), Wuhan University, 129 Luoyu Road, Wuhan, Hubei, 430079, PR China

## Abstract

HTML5 is being developed as the next major revision of HTML (Hypertext Markup Language), the core markup language of the World Wide Web. It aims at reducing the need for proprietary, plug-in-based rich Internet application (RIA) technologies such as Adobe Flash. The canvas element is part of HTML5 and is used to draw graphics using scripting (e.g., JavaScript). This paper introduces Cartagen, an open-source, vector-based, client-side framework for rendering plug-in-free, offline-capable, interactive maps in native HTML5 on a wide range of Web browsers and mobile phones. Cartagen was developed at MIT Media Lab's Design Ecology group. Potential applications of the technology as an enabler for participatory online mapping include mapping real-time air pollution, citizen reporting, and disaster response, among many other possibilities.

## Background

HTML5 is being developed as the next major revision of HTML (Hypertext Markup Language), the core markup language of the World Wide Web [1,2]. It aims at reducing the need for proprietary plug-in-based rich Internet application (RIA) technologies such as Adobe Flash, Microsoft Silverlight [3], and Oracle-Sun JavaFX [4]. For example, YouTube is planning an HTML5 version of its service that does not rely on Adobe Flash, but instead uses HTML5 to play videos in Web browsers [5]. Similarly, Apple has dropped Flash support on its iPad device [6] in favour of HTML5.

The canvas element is part of HTML5 [7] and is used to draw graphics using scripting (e.g., JavaScript). It was first introduced by Apple for use in the Mac OS X Dashboard and the Safari browser, then later implemented in Gecko-based browsers, such as Mozilla Firefox, in Opera [8], as well as in Google Chrome (Chrome is built around the same WebKit engine used in Safari). Microsoft Internet Explorer versions 7 and 8 do not yet support the canvas element out of the box, but Google Chrome Frame [9], a free plug-in for Internet Explorer, can be used to render Web pages that use HTML5 and the canvas element inside Internet Explorer.

Some HTML5 demos are available at [10], including an interesting HTML5 geolocation demo that runs on Apple iPhone OS 3, as well as in Firefox 3.5 (and later). Firefox now supports the W3C (World Wide Web Consortium) Geolocation API without the need to install any location plug-ins. It will first ask if the user wants to share her/his location. If the user agrees, it gathers information about nearby wireless access points and the user's computer IP (Internet Protocol) address then sends this information to the default geolocation service provider, Google Location Services, to get an estimate of user's location. That location estimate is finally shared with the requesting Web page, which in turn displays the user's location using Google Maps [11,12].

HTML5 and the canvas element have serious potential in many useful applications [13], but the rest of this paper will just focus on Cartagen [14], a vector-based, client-side framework for rendering maps in native HTML5, and its potential applications.

## Introduction to vector mapping and Cartagen

**Figure 1:**
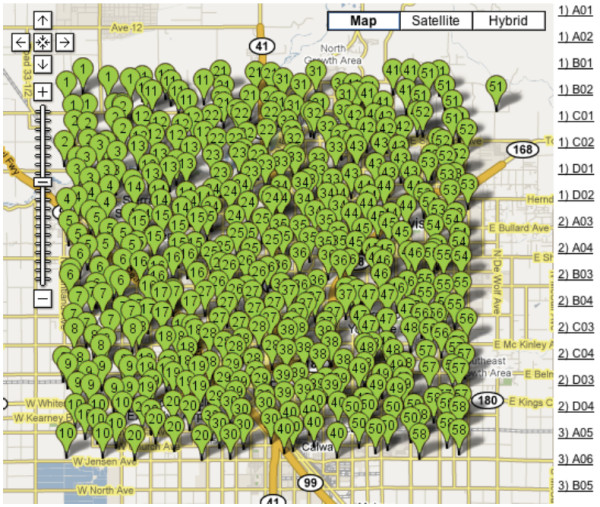
**A pincushion of unreadable dots (screenshot from **[15]**)**. The only way to negotiate this map is to zoom in, but each time the user zooms in, a new server request and server-side rendering of new map tiles must be made, which consumes more time, server resources and bandwidth.

As map data become richer and we strive to present multi-layered data in a variety of projections and map zoom levels, traditional Web mapping techniques start to become too inflexible. Most current Web maps make use of a server-side tile generator and cache system, where the desired map is rendered ahead of time and cut into image tiles at a variety of scales depending on user requests made to the server. While this works well for a single dataset, for example, terrain contours, as more data are added, the map becomes a pincushion of unreadable dots (see Figure 1 - a screenshot from [15]) until a new zoom-in level is selected by the user and rendered on the server. Using new techniques made possible by widespread browser support for HTML5 and specifically the canvas element, we can now create maps which are not pre-rendered, but generated on-the-fly. This frees us from a single projection, zoom level or representation (fetched per single server request), and enables a more dynamic, interactive, and narrative cartographic style. Discrete vector data (made up of points, lines, and areas) can be downloaded once, and displayed at any scale and in any style. Recent increases in JavaScript execution speed [16] make possible relatively high frame rates (~15 fps (frames per second) on a typical notebook), obviate the need for browser plug-ins like Flash or Java, and make dynamic HTML5 mapping accessible even on mobile devices such as on the iPhone, Android (a mobile operating system using a modified version of the Linux kernel [17]), and Windows Mobile platforms [18].

**Figure 2:**
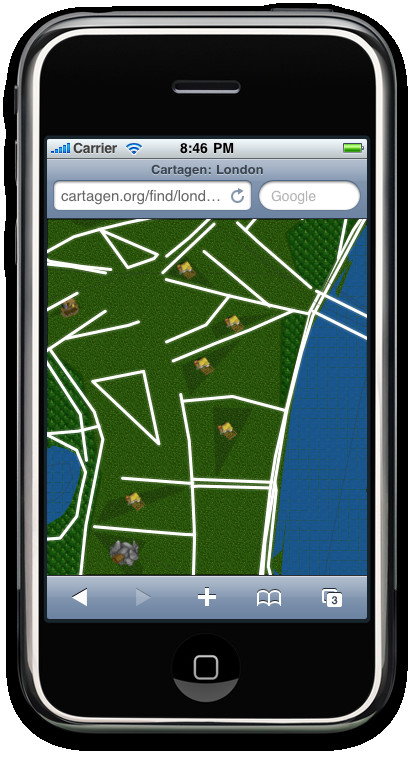
**Cartagen running on Apple iPhone**.

Instead of sending pre-rendered tiles for every zoom level, Cartagen draws maps dynamically on the client side. This means maps can move, adapt and redraw, and can include as many layers of data/levels of detail as needed. Vector mapping is done in native HTML5, which runs on the iPhone (Figure 2) and the Android platforms, and uses less bandwidth and client processing resources overall.

### Advantages of local rendering

**Figure 3:**
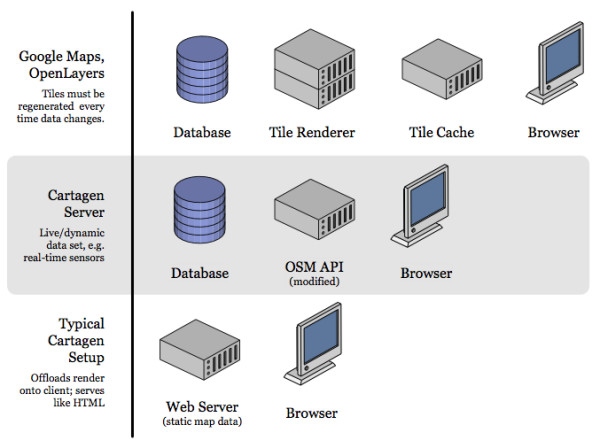
**Google Maps/OpenLayers vs. Cartagen**.

Most current in-browser maps (OpenLayers/TileCache [19], Google Maps) make use of a powerful server generating 256 × 256 pixel raster tiles in PNG (Portable Network Graphics), JPG (Joint Photographic Experts Group image format) or GIF (Graphics Interchange Format) formats, paired with a fast caching system to serve these tiles to a JavaScript or Flash-based browser display (Figure 3). Users can drag or slide the map around as if on a giant virtual piece of paper, in what is known colloquially as a 'slippy' map. This suffers from the necessity of deciding on a single map style, as tiles cannot be generated in real time without some kind of a server cluster or cloud system. Furthermore, tiles, once rendered and sent to the browser, cannot be changed - making most current Web maps "static" in this sense.

In this article we discuss the use of Cartagen, an open-source vector mapping framework developed at the MIT Media Lab's Design Ecology group [20]. Since it draws the map to the screen every frame (on the client side - Figure 3), it does not rely on a server to generate map tiles, nor is it tied to a single representational style (or server-rendered zoom levels). Multiple map datasets can be displayed simultaneously, and map features can have dynamic styles, such as different appearances on hover, click, and other user events. Maps can be dynamically labelled, scaled smoothly instead of in steps, and generally manipulated visually as desired. This makes Cartagen a much richer and more flexible map interface than what users -- and designers -- have been limited to up until now.

Cartagen was written in JavaScript and uses the new canvas element to load mapping data from various sources, including OpenStreetMap (OSM) [21]. A complete API (Application Programming Interface) and documentation for Cartagen can be found at [22].

### GSS and vector data

With our newfound ability to change how a map is displayed comes the need for a means to express map styles, and Cartagen takes as its inspiration a well-known means of styling on the Web: CSS, or Cascading Style Sheets. In Cartagem maps are styled with Geographic Style Sheets (GSS), a cascading stylesheet specification for geospatial information that leverages CSS to make map styling more accessible. GSS is also a scripting language, making Cartagen an ideal framework for mapping dynamic data. (The use of CSS-like syntax to style maps was first demonstrated by Michal Migurski's Cascadenik [23], which generated Mapnik style documents based on a CSS-like input format that was far easier to write by hand. Following Cartagen's release, the OpenStreetMap Potlatch renderer began using a similar syntax called MapCSS [24].)

Those familiar with the content-style division between HTML and CSS will find a similar distinction between Cartagen's map data format (OSM-JSON or OpenStreetMap-JavaScript Object Notation [25]) and its style language, Geographic Stylesheets, or GSS.

GSS is a simple way to associate map styles such as line width, fill colour, font size and family, and a wide array of other aesthetic factors with either specific map elements or more commonly with tags such as 'park' or 'highway'. A simple GSS style might look like this:

   park: {

      strokeStyle: "green",

      lineWidth: 3,

   }

This would represent any feature tagged as a 'park' with a 3px green border. GSS goes far beyond simple colours, however, as we will demonstrate here.

## Setting up a Cartagen map

Cartagen is available for download at [26]. The standard download ('cartagen-client-0.6.2.zip' - filename of the current version at the time of writing) consists of an 'index.html' Web page upon which the map is displayed, and a 'cartagen.js' file (240 kilobytes) in the 'cartagen' directory which contains all the framework code. It also contains substantial sample map data (from OpenStreetMap; 'CC By SA'-- Creative Commons Attribution-Share Alike license) for the city of Rome, which will load if you open the 'index.html' file in Firefox, Safari, Chrome, or Opera. (If you are using Windows or Linux, you may have to unpack the zip file before opening any of its contents in a browser; you will not be able to run Cartagen from within the zip file itself.)

Cartagen reads map data in (among other formats) JavaScript Object Notation, or JSON [25], a common standard for structured data which is similar to XML (Extensible Markup Language). JSON files, commonly identifiable by their '.json' file extension, are natively parse-able by modern JavaScript-enabled browsers and are more human-readable than XML. A simple map feature might be represented as:

   {"osm":

      {"node":

         [

            {"lat":"42.3608",

               "lon":"-71.08768",

               "visible":"true",

               "display":true,

               "img":"image-url.png"

            },

            {"lat":"42.3608",

               "lon":"-71.08768",

               "visible":"true",

               "display":true,

               "img":"image-url.png"

            }

         ]

      }

   }

Cartagen follows the OpenStreetMap (OSM) data model of nodes, ways, and relations, and this 'OSM-JSON' is a fairly literal translation from OpenStreetMap's XML data format. (OSM-JSON exhibits several important advantages over the GeoJSON standard [27]; by associating nodes and ways by reference, polygons which share many nodes, such as shared borders between two countries, need not have redundant node definitions. Instead they simply reference a set of shared nodes by their node IDs. In addition, OSM-JSON, like OSM-XML, can be served from a database with minimal resources since it mirrors the database structure so closely, essentially offloading the polygon assembly onto the client.) We will learn soon how to manipulate and create your own datasets, but for starters, let us grab some data from the OpenStreetMap dataset.

In a new browser window, open Cartagen's main homepage [14], type any place name into the 'Go somewhere' box, e.g., 'Shanghai', and press Go. You should see the desired location load, and if there is a reasonable amount of data available, a map will be drawn as you watch. To download this data, press 'Download data »' at the lower left corner of the page, and you will be prompted to select a region. Unless you have a very powerful computer, just select some small area (say, a few kilometres wide).

**Figure 4:**
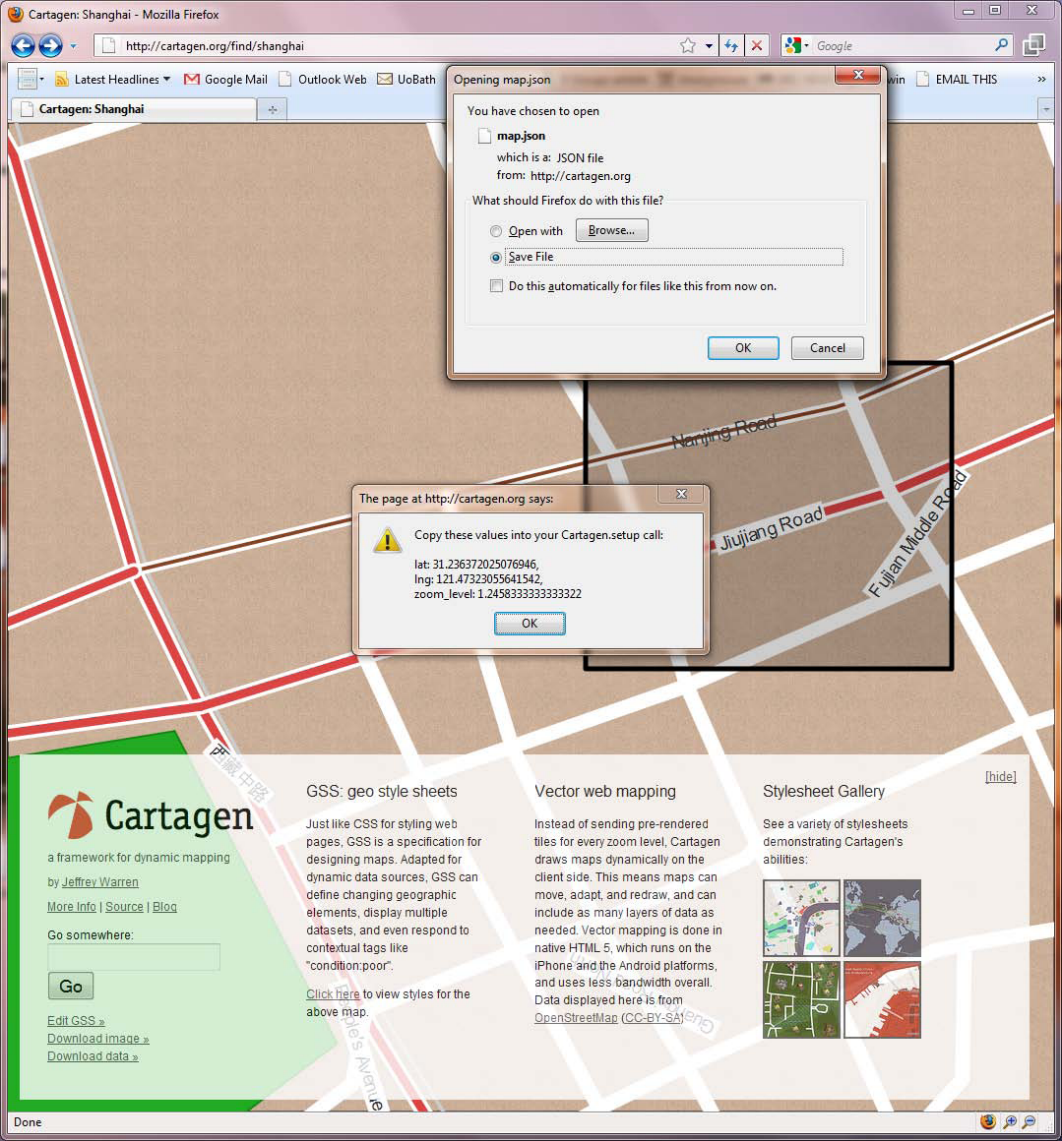
**Downloading data from a map of Shanghai, China, in Cartagen**. Note the 'Download data' link (lower left).

A window will appear with the bounding coordinates of your selection (Figure 4). Copy these coordinates down in the format provided; we will need them in a moment. The data will also begin downloading immediately in a file called 'map.json' behind the current browser window. Go to your 'downloads' folder and move that file into the 'cartagen-client-0.6.2' folder we downloaded earlier.

Now, open the 'index.html' file in a text editor, like TextMate on the Mac, Notepad++ on Windows, or Emacs or Vim on Linux. (Mac users may have trouble viewing the raw code with TextEdit, and Windows users may have trouble with carriage returns in Notepad. A professional text editor is preferable.) You will see, a few lines down, the following setup code, which initialises the Cartagen environment:

   <script type="text/javascript" charset="utf-8">

      Cartagen.setup({

         stylesheet: "samples/rome/style.gss",

         static_map: true,

         static_map_layers: [

            "samples/rome/park.js",

            "samples/rome/rail.js",

            "samples/rome/waterway.js",

            "samples/rome/primary.js",

            "samples/rome/secondary.js",

            "samples/rome/building.js",

            "samples/rome/area.js",

            //"more.json"

         ],

         lat: 41.891,

         lng: 12.4902

      })

   </script>

Go ahead and remove the static_map_layers lines, and in their place, add a single static_map_layer called 'map.json' - this will load in the new data we just downloaded, assuming you placed the file in the Cartagen root folder correctly. You should be left with:

   <script type="text/javascript" charset="utf-8">

      Cartagen.setup({

         stylesheet: "samples/rome/style.gss",

         static_map: true,

         static_map_layers: [

            ***"map.json"***

         ],

         lat: 41.891,

         lng: 12.4902

      })

   </script>

Next, find the last two lines of setup, specifying 'lat' and 'lng' (latitude and longitude). Replace them with the code we copied down earlier when we downloaded new data (Figure 4), so that we may end up with something like this (new code in bold-italics):

   <script type="text/javascript" charset="utf-8">

      Cartagen.setup({

      stylesheet: "samples/rome/style.gss",

      static_map: true,

      static_map_layers: [

         ***"map.json"***

      ],

      ***lat: 31.236372025076946***,

      ***lng: 121.47323055641542***,

      ***zoom_level: 1.24***

      })

   </script>

In the above example, we downloaded a chunk centred around parts of Nanjing Road and Jiujiang Road, Shanghai, China, so this code now ensures that the map is centred on that selection rather than the default Rome dataset, and the zoom_level we were using is also preserved. Reload the 'index.html' page in your browser, and you should see the area you selected. (If you do not, be sure you have preserved all the commas, brackets, and other formatting.) This map is completely local; it is not making any calls to distant tile servers, and if disconnect from the Internet, it will still work. Put these files on a server, and without a tile server or caching system, you have yourself a map.

Now let us get rid of any other stuff we do not want, such as the big Cartagen overlay with the logo and such. All we really need on the page are the following lines inside the <head> tags:

   <link rel="stylesheet" href="style.css" type="text/css" media="screen" title="no title" charset="utf-8">

   <script src="cartagen/cartagen.js" type="text/javascript" charset="utf-8"></script>

   <script type="text/javascript" charset="utf-8">

      Cartagen.setup({

         // your setup code here

      })

   </script>

And just one line in the <body> tags:

   <canvas id="canvas"></canvas>

### Styling the map with GSS

Now we are ready to change the map's appearance with our own stylesheet. Let us begin by looking at the Cartagen.setup() code we have already. The very first line defines what stylesheet to use with this data: 'stylesheet: "samples/rome/style.gss"'. To use our own, we simply need to create a new file called 'somefilename.gss' in the root directory of Cartagen and change the Cartagen.setup() line to point to our new stylesheet. In ' somefilename.gss', let us start with the basic styles:

   body: {

      pattern: "samples/rome/images/brown-paper.jpg",

   },

   way: {

**Figure 5:**
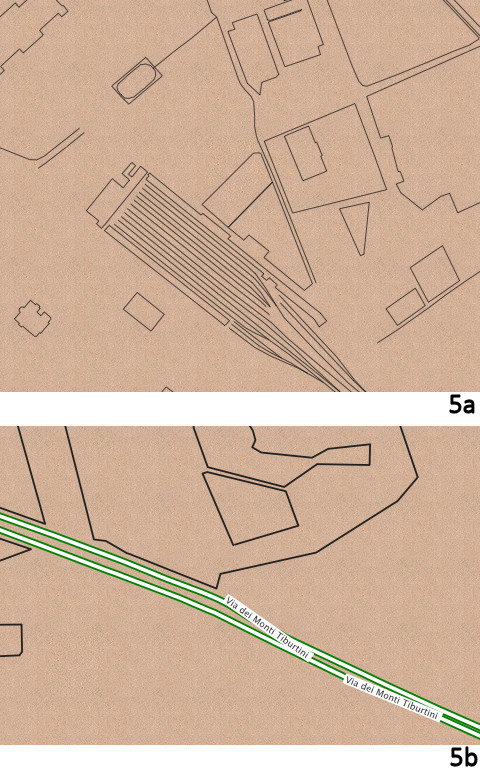
**Experimenting with GSS in Cartagen**.

      strokeStyle: "#222",

      lineWidth: 3,

   },

These define the background colours and the default style for any 'way' or line. You should see a brown paper background colour with fine dark grey lines on it (Figure 5a). Notice that we are using hexadecimal colour codes ('#222') just as in CSS, though we can also use words like 'green' or 'rgba(0.1,1,0.8,0.4)' for full RGB (Red Green Blue) colour with an additional alpha (A) channel.

Next let us add a new style for map features tagged as 'building':

   building: {

      fillStyle: "#444",

      opacity: 0.4,

   }

Here is a more complex style (Figure 5b):

   highway: {

      strokeStyle: "white",

      lineWidth: 6,

      outlineWidth: 3,

      outlineColor: "green",

      fontColor: "#333",

      fontBackground: "white",

      fontScale: "fixed",

      text: function() {

         return this.tags.get('name')

      }

   }

A full list of possible styles is available at [28].

### Adding new data

Displaying streets and parks is not enough; we need to be able to display our own data. To do this, we can add our own Nodes and Ways. (OSM terminology, which Cartagen follows, uses Nodes and Ways instead of the more common 'points' and 'polygons', respectively.) Let us add a set of points with custom icons. First, we will create a file in the Cartagen root directory called 'mine.json'. Let us say we have four geographic locations and we want to mark each with a unique icon. In 'mine.json', we can add them in the following format:

   {"osm":

      {"node":

         [

            {"lat":"42.3608",

               "lon":"-71.08768",

               "visible":"true",

               "display":true,

               "img":"image-url.png"

            },

            {"lat":"42.3608",

               "lon":"-71.08768",

               "visible":"true",

               "display":true,

               "img":"image-url.png"

            }

         ]

      }

   }

**Figure 6:**
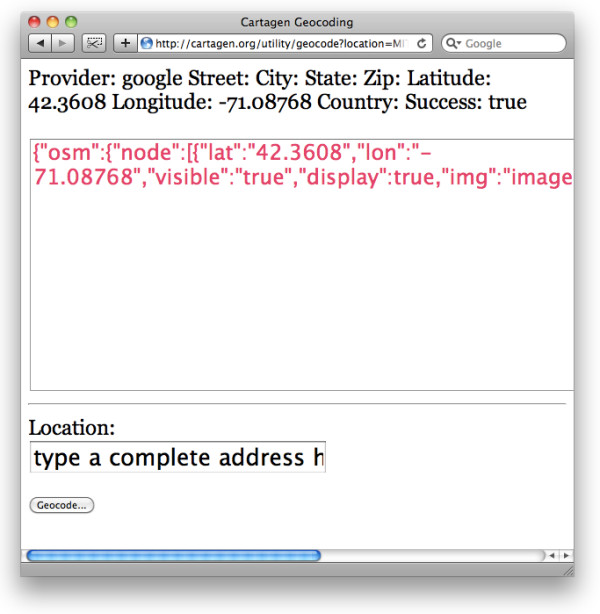
**Cartagen geocoding service returns well-formatted OSM-JSON statements**.

To make finding the latitude and longitude of our locations easier, there is a service at [29] which, if you type in an address, like '31 Maple Ave, Cambridge, Massachusetts', will return a well-formatted OSM-JSON statement (Figure 6).

To add a polygon, we will need to create a set of nodes, and then reference them in a 'way' feature. Cartagen will look for each of the constituent nodes of the way and connect them into a polygon. This tends to look a little complicated:

   {"osm":

      {"node":

         [

            {"lon": "12.5103451",

               "id": "339420390",

               "lat": "41.8569274"},

            {"lon": "12.5106756",

               "id": "339420395",

               "lat": "41.8569569"},

            {"lon": "12.5107474",

               "id": "339420398",

               "lat": "41.8565097"},

            {"lon": "12.5104169",

               "id": "339420392",

               "lat": "41.8564803"}

         ],

      "way":

         [

            {"visible": "true",

               "nd":

                  [

                     {"ref": "339420390"},

                     {"ref": "339420395"},

                     {"ref": "339420398"},

                     {"ref": "339420392"},

                  ],

               "tag":

                  [

                     {"v": "Fosse Ardeatine", "k": "name"},

                     {"v": "catholic", "k": "denomination"},

                  ]

         ]

      }

   }

The above polygon outlines a building in Rome, and was drawn from the OpenStreetMap dataset. To make things easier, the next version of Cartagen will have a graphical polygon-drawing tool which will return an OSM-JSON representation of anything the user can draw. Support for other data formats, such as GeoJSON, is also in progress.

**Figure 7:**
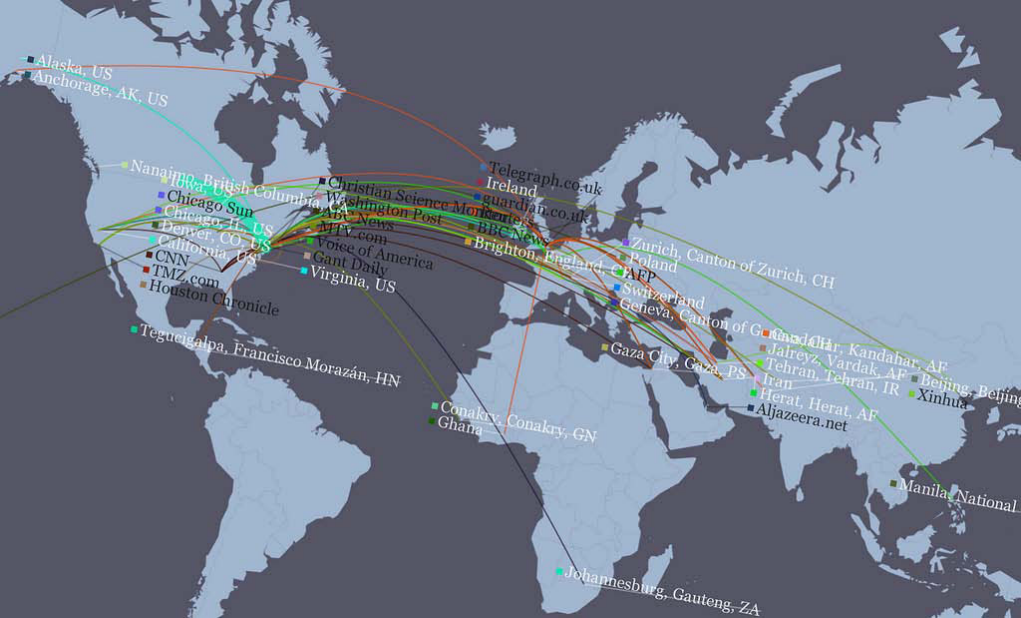
**Screenshot of NEWSFLOW**. Built with HTML5 on the dynamic mapping framework of Cartagen, NEWSFLOW is a dynamic, real-time interactive map of news reporting, which displays both the latest top stories as well as the news organisations/agencies that covered them. Arcs link the location of the news organisations' headquarters to the places mentioned in a given news article.

If one can write JavaScript, one could periodically update the positions of these data points; they are native JavaScript objects. A more advanced map example created with Cartagen is available at [30] (Figure 7), in which news stories fetched every minute from Google News are painted onto an interactive map of the world, where the user can explore news by topic or region.

### Advanced techniques: context menus and scripting

**Figure 8:**
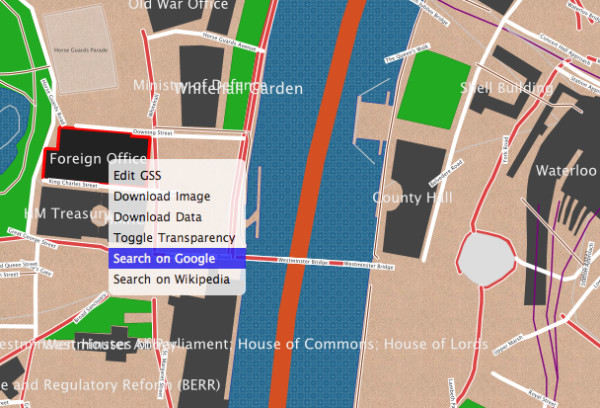
**A right-click context menu in Cartagen**.

Let us try some more advanced features, including right-click contextual menus. Cartagen has built-in support for context menus (Figure 8); to add one to a particular feature, we have to modify the appropriate GSS style:

   building: {

      fillStyle: "#444",

      text: function() { return this.tags.get('name') },

      hover: {

      fillStyle: '#222'

   },

   menu: {

      "Show me the name": function() {

            alert(this.tags.get('name')

         },

      "Show me the code": function() {

      window.open("http://en.wikipedia.org/wiki/Special:Search?go=Go&search=" + this.tags.get('name'), "_blank")

         }

      }

   },

We have done a couple of things here. First, let us look at the 'text' attribute, where instead of a bit of text like 'Building', we have written "function() { return this.tags.get('name') }". This is a simple JavaScript function that returns "this.tags.get('name')". The 'this' keyword is used here to access the map feature being displayed. We then look in that feature's tags and 'get' the tag called 'name'. The end result is that all map features tagged as 'building' are labelled with their name. There are a number of attributes in the 'this' object, such as 'this.area' which yields the x, y area of the polygon (if applicable), 'this.author'- yielding the OpenStreetMap username who created the feature, 'this.lat', 'this.lon', and more.

Notice that we have done the same thing with one of the menu items lower down. There are two menu items, one called 'Show me the name' and one 'Show me the code'. Each will pop up a standard JavaScript alert if the user right clicks on the building and selects them. The first should be familiar; it just displays the name of the building (assuming it has one), using the same syntax as the 'text' attribute.

The second menu item is a bit more interesting. It actually grabs the name of the building and creates a Wikipedia search for that name in a new browser window (Figure 8). If there is no name, this will search for 'null' or fail, so it is somewhat sloppy code, but still gives the reader a taste of the kind of scripting one can achieve with Cartagen, and of the kinds of data that are available to developers in the map. By contrast, tile-based maps usually have no metadata on a per-feature basis, and no means for associating metadata with specific features.

## Discussion

Cartagen is perhaps the first comprehensive dynamic mapping framework of its kind to leverage HTML5 and the canvas element for plug-in-free, offline-capable interactive online mapping. More recently, Google demoed their first fully HTML5-based version of Google Maps on a Palm Pre (a multimedia smartphone running a Linux-based operating system) [31]. The ability to build applications that run natively in the browser without the need for plug-ins will allow agencies such as the police, government, and health services, to view data that are currently restricted due to rules on installing plug-ins and software on networks. It will also enable those agencies to avoid single vendor's proprietary technologies from Adobe (Flash), Microsoft (Silverlight) and others [32]. Such technologies are also reportedly known to consume more processing resources, leading to significantly shorter battery life on smartphones and other mobile devices, compared to pure HTML5 content.

Performance wise, JSON [25] is reported to be faster and more efficient than GML (Geography Markup Language, an XML grammar defined by the Open Geospatial Consortium (OGC) to express geographical features [33]) [34]. (Cartagen uses OSM-JSON, which is supposed to be even more efficient than GeoJSON [27].) Moreover, compared to SVG (Scalable Vector Graphics) maps [35], canvas seems to cope better with large numbers of rendered objects [36]. Additional canvas performance tests are available at [37]. Performance is expected to further improve with time, as the HTML5 specification gets finalised and Web browser implementations of it become fully optimised and mature, (though the road ahead might not be so smooth [38,39]).

### Applications potential of HTML5 maps

#### Live data streams and accessible participatory mapping by the masses

Cartagen can display maps that change based on live data streams. For example, Cartagen can show live OpenStreetMap data whereby viewers see edits occurring in real time, with no rendering load on the server. Users are enabled and empowered to create their own maps - not just pushpins and overlays, but completely designed maps that incorporate rich and dynamic data and tell unique stories. Instead of a single canonical map for everyone, individuals and communities can now much more easily make locally and personally relevant maps. This is particularly important in today's era of participatory GIS (Geographic Information Systems) and social Web ("Web 2.0") mapping, to create more empowered and active communities that are fully involved in the civil society and in the management of different aspects of the local and global environments at ordinary times as well as during times of emergency, e.g., active citizens' involvement in disaster reporting and management, etc.

Cartagen can potentially become an enabler of community participation by means of SMS (Short Message Service) mapping and search. String-based geocoding (e.g., 'map Bhagalpur, India') allows users to produce their own maps from in the field with only a basic cell phone. This can widen participation to more than four billion cell phone users worldwide, as well as to rural regions outside the reach of cable and landline-telephone-based Internet (SMS does not even need a mobile Internet connection to report a mappable incident). Geographic mapping with text messages has applications in incident reporting by citizens, in disaster response, and in many health and healthcare scenarios (*cf*. Depiction [40]).

#### Business data mapping on the Virtual Globe background layer

The emergence of Virtual Globe software systems such as Google Earth, Microsoft Virtual Earth, and NASA World Wind has revolutionised the traditional way of using geospatial information, making global geospatial information easily accessible and readily usable by the general public instead of only the highly-skilled domain experts [41]. The wide popularity of these Virtual Globe software systems in the geospatial and general communities has opened up many more ways of exploring and using them in various aspects of everyday life. One potential scenario in the context of this paper is to combine the dynamic rendering power of HTML5 with the excellent high-quality and high-resolution background maps and imagery provided by Virtual Globes. Thus users can overlay their operational data on the Virtual Globe background layer in their business context and have a more intuitive experience to interpret the data. The dynamic, interactive, and narrative cartographic style enabled by HTML5 is suitable for the representation of the dynamic and light-weight vector data, while Virtual Globe technology is appropriate for digestion of Web-based, large-scale geospatial information and raster imagery. Therefore, HTML5 and Virtual Globe technologies could be complementary, providing comprehensive geospatial data, which are either dynamic or static, and richer experiences to users.

#### Spatial analysis based on dynamic data

The primary focus of using HTML5 in the context of this paper is dynamic cartographic mapping. Such visualisation functions can be combined with conventional spatial analysis functions, thus allowing users to investigate geoscientific processes. For example, a geospatial buffer analysis function can be used when evaluating the influence of flood hazard, and the analysis result can then be visualised on-the-fly. The incorporation of data analysis functions requires exposing conventional spatial analysis functions over the Web. A service-oriented paradigm can be employed, where traditional siloed geospatial analysis functions are exposed as Web Services with interoperable interfaces and protocols. In the geospatial domain, there is already a standard focusing on geoprocessing over the Web, namely OGC Web Processing Service (WPS) specification [42]. Standards-based interoperable geoprocessing services make a large amount of geospatial analysis functionalities easily accessible to users, much like their local resources are, and allow for plug-and-play analysis of geospatial data. Vector data visualised in Cartagen can be sent to geoprocessing services for on-demand analysis and the results then fed back to Cartagen to be layered over existing data. The introduction of geospatial analysis functions and Web Service technologies will significantly extend and expand the original mapping goals of Cartagen, making it more powerful and scalable in addressing the analysis demands of front-end users.

## Conclusions

In this paper, we have demonstrated a variety of interactive HTML5 Cartagen map features which would have been impossible or much more complicated to realise using conventional tile-based online mapping systems: per-feature hover and click events, per-feature style definitions, offline generation of Web map imagery, and full access to map geometry and feature metadata through the 'this' keyword. In addition, the use of map tags via the CSS 'class' convention offers a flexible and easily editable means to author new styles and generate new maps. Ultimately, dynamic and scriptable map environments such as Cartagen/GSS will change how maps are perceived and used; instead of merely reading maps, more and more users will be encouraged to interact with, modify, and remix geographic information, all without requiring any special browser plug-ins to view or share their creations, which is of critical importance in those environments where restrictions are in place on installing plug-ins and network software. We see Cartagen as a first step towards such a participatory cartographic HTML5 medium for communication.

## Competing interests

The authors declare that they have no competing interests.

## Authors' contributions

MNKB researched the topic background and its strengths and potential applications, and conceived and drafted the manuscript. JW, the developer of Cartagen, provided first-hand information and tutorial text (including sample code) about Cartagen, and also contributed all the figures in this paper, except Figure 4, which was prepared by MNKB. JG and PY contributed text about the applications potential of HTML5 maps, as well as a number of valuable suggestions that helped improve other parts of the paper. All authors read and approved the final manuscript. Commercial products and company/brand names mentioned in this paper are trademarks and/or registered trademarks of their respective owners.
